# The First Quarterly Clinic of the Maryland State Dental Society

**Published:** 1894-11

**Authors:** 


THE FIRST QUARTERLY CLINIC of the Maryland State Dental Society, was given at the Eutaw House, Baltimore, Saturday, October 13, 1894, at 2 P. M. Evening Session at 8 P. M.

A feature of the evening was the attendance of about thirty dentists from Washington. The following is the programme.

1. Exhibition of Models and Explanation of Procedure in Correcting Irregularity. A. W. Sweeny, D. D. S., Washington D. C.

2. Clinic. W. G. A. Bonwill, D. D. S., Philadelphia, Pa.

3. Uniting Gold and Tin in filling Teeth. Henry Bliss Noble, D. D. S., Washington D. C.

4. Clinic. T. S. Waters, D. D. S., Baltimore.

5. Abbey's Soft Foil in filling Teeth. Cyrus M. Gingrich, D. D. S., Baltimore.

6. Method of Working Crystal Mat Gold with hand pressure only. H. M. Schooley, D. D. S., Washington, D. C.

7. Clinic. A Lee Penuel, D. D. S., Leesburg, Va.

8. Methods of Correcting Irregularities. C. C. Harris, M. D., D. D. S., Baltimore.

9. Strengthening the Spyer's Method of Suction Plates. David Genese, D. D. S., Baltimore.

10. Clinic. Porcelain Inlays. B. Myer D. D. S., Baltimore.

